# Efficacies of prevention and control measures applied during an outbreak in Southwest Madrid, Spain

**DOI:** 10.1371/journal.pone.0186372

**Published:** 2017-10-13

**Authors:** Anaiá da Paixão Sevá, Maia Martcheva, Necibe Tuncer, Isabella Fontana, Eugenia Carrillo, Javier Moreno, James Keesling

**Affiliations:** 1 Department of Mathematics of University of Florida, Gainesville, Florida, United States of America; 2 Department of Mathematical Sciences of Florida Atlantic University, Boca Raton, Florida, United States of America; 3 Ministry of Agriculture, Livestock and Food Supply of Brazil, Brasília, Distrito Federal, Brazil; 4 WHO Collaborating Centre for Leishmaniasis, Centro Nacional de Microbiologia, Instituto de Salud Carlos III, Majadahonda, Madrid, Spain; Tulane University, UNITED STATES

## Abstract

Leishmaniasis is a vector-borne disease of worldwide distribution, currently present in 98 countries. Since late 2010, an unusual increase of human visceral and cutaneous leishmaniasis cases has been observed in the south-western Madrid region, totaling more than 600 cases until 2015. Some hosts, such as human, domestic dog and cat, rabbit (*Oryctolagus cuniculus*), and hare (*Lepus granatensis*), were found infected by the parasite of this disease in the area. Hares were described as the most important reservoir due to their higher prevalence, capacity to infect the vector, and presence of the same strains as in humans. Various measures were adopted to prevent and control the disease, and since 2013 there was a slight decline in the human sickness. We used a mathematical model to evaluate the efficacy of each measure in reducing the number of infected hosts. We identified in the present model that culling both hares and rabbits, without immediate reposition of the animals, was the best measure adopted, decreasing the proportion of all infected hosts. Particularly, culling hares was more efficacious than culling rabbits to reduce the proportion of infected individuals of all hosts. Likewise, lowering vector contact with hares highly influenced the reduction of the proportion of infected hosts. The reduction of the vector density per host in the park decreased the leishmaniasis incidence of hosts in the park and the urban areas. On the other hand, the reduction of the vector density per host of the urban area (humans, dogs and cats) decreased only their affected population, albeit at a higher proportion. The use of insecticide-impregnated collar and vaccination in dogs affected only the infected dogs’ population. The parameters related to the vector contact with dog, cat or human do not present a high impact on the other hosts infected by *Leishmania*. In conclusion, the efficacy of each control strategy was determined, in order to direct future actions in this and in other similar outbreaks. The present mathematical model was able to reproduce the leishmaniasis dynamics in the Madrid outbreak, providing theoretical support based on successful experiences, such as the reduction of human cases in Southwest Madrid, Spain.

## Introduction

Leishmaniasis is a vector-borne infectious disease of worldwide distribution, occurring in 98 countries. Annually, there are 900,000–1.3 million new cases and 20,000 to 30,000 deaths [1]. Compared to the cutaneous leishmaniasis form (CL), visceral leishmaniasis (VL) is more severe and can be fatal in humans and dogs [2]. In Spain, it is an endemic zoonosis caused by *Leishmania infantum*, which is transmitted by the sandfly *Phlebotomus perniciosus* (Diptera: Psychodidae: Phlebotominae) bites [3].

Since late 2010, an unusual increase of human leishmaniasis cases (VL and CL) has been observed in the south-western Madrid region [4]. The number of human cases of cutaneous and visceral leishmaniasis increased from 27 (2009–2010) to 172 (2011) and latter to 204 (2012) [5]. Among these cases, 36.5% were VL and 63.5% were CL [5,6]. Residents of Fuenlabrada, Leganés, Getafe, and Humanes de Madrid municipalities of the south Madrid metropolitan area have been affected [4]. Since the beginning of this outbreak, the population of these four municipalities exceeds half a million people [6].

Conditions that could favor the higher incidence of the disease can be associated with a higher density of infected vectors and the emergence of new reservoirs in the area, both consequences of changes in environmental and climatic conditions [7].

Domestic dogs are natural reservoirs for *L*. *infantum* and in other countries they actually are the most important cause of human VL spread in endemic areas [8]. VL is considered a disease of both veterinary and public health importance [9], since in endemic areas for *L*. *infantum*, increased incidence of canine leishmaniasis has occurred prior to the appearance of human cases of VL [10].

Dog’s seroprevalences in the Mediterranean basin ranges from 5% to 30% depending on the region [11]. Interestingly, in this outbreak, the dog VL seroprevalence during the same period and in the same area, was found to be only 1.64% [12]. Seropositivity for *Leishmania* was found also in other species, such as cats (*Felis catus*) (2.3%) and rabbits (*Oryctolagus cuniculus*) (17.1%) [13]. However, the most interesting result was found in hares (*Lepus granatensis*), as 43 of the 148 (29%) analyzed animals were parasite positive. This fact was reported in a research conducted between December 2011 and July 2012 [6,14], which concluded that the strains found in hares and humans are the same [15]. Hares also showed capacity to infect the sandfly vector [12]. In addition, studies conducted from 2009 to 2012, in Madrid, indicated a leishmaniasis seroprevalence of 31.8% in hares, and a greater percentage of *L*. *infantum* DNA positivity by PCR analysis in hares (43.5%) than in rabbits (8.6%) [13]. Therefore, all these data obtained suggest that hares might be considered an important reservoir of the parasite in this outbreak [13,15]. The association with periurban green areas indicated that the urban transmission cycle was dependent upon a relationship with the sylvatic environment [15].

Because of the hare role in this outbreak, one of the measures adopted to avoid transmission and appearance of new cases of human leishmaniasis was to reduce the reservoir presence by capturing hares and rabbits. Another measure adopted by the Community of Madrid for the disease control was to reduce the amount of vectors by using insecticides in the green park [14], hence modifying the habitat to hinder the biological cycle. These measures were being redefined according to the outbreak progress, and resulted in a slight sickness reduction in 2013, with respect to the three previous years. Thus, in 2013, 2014, and 2015 there were 90, 92, and 39 human VL cases, respectively. However, other alternative measures have been used, as vaccine and insecticide-impregnated collars in dogs, even though the seroprevalence was low in these animals during this outbreak.

While these strategies have been at present successful in preventing and controlling the disease in this outbreak of leishmaniasis, we aimed to understand the effectiveness of each strategy employed singly. Thus we used a mathematical model to evaluate their impact in all populations involved, as well as on the reduction of infected hosts. This mathematical model has been previously proposed [16] and in this case it has been specifically adopted to the outbreak of Madrid. This study provides a theoretical support based on successful experiences in leishmaniasis prevention and control programs in zoonotic VL areas in order to direct future actions in this and in other similar outbreaks.

## Methodology

### Mathematical model

**Fig 1:**
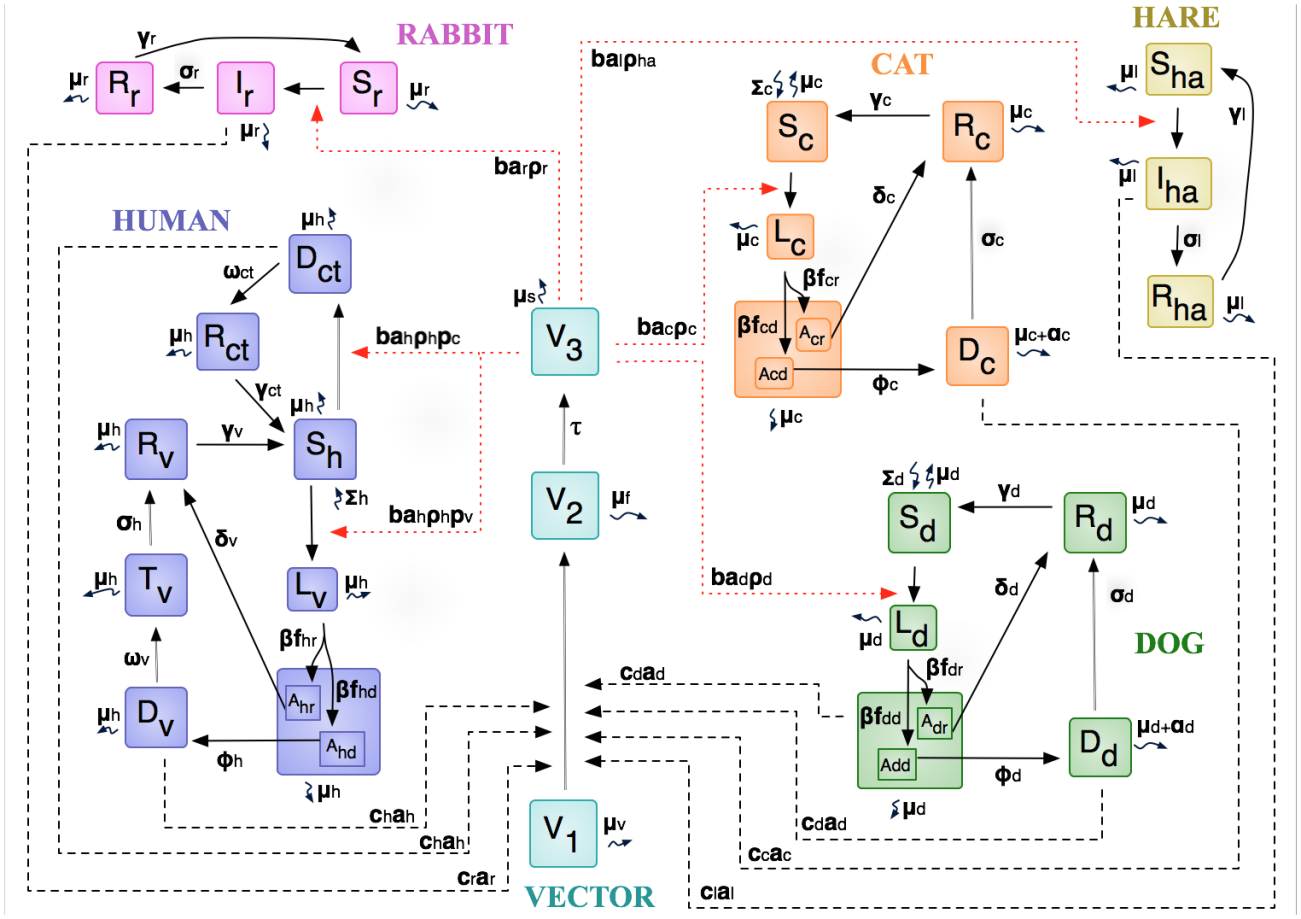
Model of the leishmaniasis dynamic. Vector: V1) Non infected, V2) Infected but not infective; V3) Infected and infective; Human: S_h_) Susceptible; D_ct_) Sick with cutaneous leishmaniasis; L_v_) Latent with visceral leishmaniasis, A_hr_ and A_hd_) Asymptomatic; D_v_) Sick with visceral leishmaniasis; T_v_) Visceral leishmaniasis in treatment; R_c_ and R_vc_) Recovered; Dogs and Cats: S) Susceptible; L) Latent with visceral leishmaniasis, A) Asymptomatic; D) Sick with visceral leishmaniasis; R) Recovered; Hares and Rabbits: S) Susceptible; I) Infectious; R) Recovered.

Research conducted in the outbreak area has shown that the vectors feed on rabbits, hares, dogs, cats, and humans [17]. Taking these findings into account, these five hosts and the vector were included in a differential equation mathematical model, which is an adaption of the model of Sevá et al. [16]. We assumed a constant population size for the vector and all hosts. The dynamics of the disease is shown in the model diagram of Fig 1.

### Natural history of the disease

#### Humans

Humans are born as susceptible (S_h_) at a *per capta* birth rate of **Σ**_h_, which consists of the sum of both other causes of death and visceral leishmaniasis caused death rates (**μ**_h_ and **α**_**h,**_ respectively) in all human populations, assuming a constant population size.

Susceptible individuals (S_h_) are infected at a rate **ba**_**h**_**ρ**_**h**_**p**_**c**_ or **ba**_**h**_**ρ**_**h**_**p**_**v**_, where **b** is the probability of becoming infected when bitten by an infective sandfly (V_3_), **a**_**h**_ is the daily average of the biting rate on humans, **ρ**_h_ is the density of the vectors per human, and **p**_**c**_ and **p**_**v**_ are the individuals’ fractions that evolve to CL and VL, respectively (Eq 1).

Cutaneous leishmaniasis: After becoming infected, humans develop the cutaneous lesions characteristic of the disease and are included in the D_ct_ compartment, staying there from the onset of the symptoms until the end of hospital treatment. At a rate **ω**_ct_, treated individuals go to R_ct_ compartment, characterized by having high immune response and not being able to infect the vector or become infected. When the cellular immune response decreases to zero, at a rate **γ**_**ct**_, recovered (R_ct_) individuals become susceptible again, going to the S_h_ compartment (Eqs 2 and 3).

Visceral leishmaniasis: After infection, humans enter the early asymptomatic stage L_v_, where they are not able to infect the vectors. Seroconversion is characterized by two late asymptomatic stages (A_hr_ and A_hd_), and occurs at a rate **β**. A fraction **f**_**hr**_ of latent individuals will recover spontaneously after going through the asymptomatic stage A_hr_, and another minor fraction **f**_**hd**_ move to the symptomatic stage A_hd_. The A_hr_ individuals stay at this compartment until their humoral immune response becomes low or reaches zero, at a rate **δ**_**v**_, and then they go to the recovered compartment (R_v_). The A_hd_ individuals become sick at a rate **ϕ**_**h**_, going to the D_v_ compartment, where they can infect the vector and die at a rate **α**_**h**_. After treatment, the sick individuals move to the T_v_ compartment, at a rate **ω**_v_, where they are still able to infect the vector. When the humoral immune response becomes low or reaches zero, at a rate **σ**_**h**_, treated individuals go to the R_v_ compartment, characterized by high cellular immune response and not being able to infect the vector or to become infected. When recovered individuals lose their cellular immune response they become susceptible again going to the S_h_ compartment, at a rate **γ**_**v**_ (Eqs 4–9).

$$\frac{dS_{h}}{dt}=-S_{h}V_{3}ba_{h}\rho_{h}\left(p_{c}+p_{v}\right)+R_{v}\gamma_{v}+R_{ct}\gamma_{ct}+{\text{Σ}}_{h}$$ (1)

$$\frac{dD_{ct}}{dt}=S_{h}V_{3}ba_{h}\rho_{h}p_{c}-D_{ct}\left(\omega_{ct}+\mu_{h}\right)$$ (2)

$$\frac{dR_{ct}}{dt}=D_{ct}\omega_{ct}-R_{ct}\left(\gamma_{ct}+\mu_{h}\right)$$ (3)

$$\frac{dL_{v}}{dt}=S_{h}V_{3}ba_{h}\rho_{h}p_{v}-L_{v}\left(\beta f_{hr}+\beta f_{hd}+\mu_{h}\right)$$ (4)

$$\frac{dA_{hr}}{dt}=L_{v}\beta f_{hr}-A_{hr}\left(\delta_{h}+\mu_{h}\right)$$ (5)

$$\frac{dA_{hd}}{dt}=L_{v}\beta f_{hd}-A_{hd}\left(\phi_{h}+\mu_{h}\right)$$ (6)

$$\frac{dD_{v}}{dt}=A_{hd}\phi_{h}-D_{v}\left(\omega_{v}+\mu_{h}+{\text{α}}_{\text{h}}\right)$$ (7)

$$\frac{dT_{v}}{dt}=D_{v}\omega_{v}-T_{v}\left(\sigma_{h}+\mu_{h}\right)$$ (8)

$$\frac{dR_{v}}{dt}=T_{v}\sigma_{h}+A_{hr}\delta_{h}-R_{v}\left(\gamma_{v}+\mu_{h}\right)$$ (9)

#### Dogs and cats

These hosts are born as susceptible (S_d_ and S_c_ for dogs and cats, respectively) at a *per capta* birth rate of **Σ**_**d**_ or **Σ**_**c**_, which consists of the sum of both leishmaniasis caused (**α**_**d**_ and **α**_**c**_, for dogs and cats, respectively) and natural (**μ**_**d**_ and **μ**_**c**_, for dogs and cats, respectively) death rates of the dog populations, assuming a constant population size. Susceptible individuals are infected at a rate **baρ**, where **b** is the probability of becoming infected when bitten by an infective sandfly (V_3_), **a**_**d**_ and **a**_**c**_ are the daily averages of the biting rates on dogs and cats, respectively, and **ρ**_**d**_ and **ρ**_**c**_ are the density of the vectors per dog and cat, respectively. After infection, hosts enter an early asymptomatic stage L_d_ or L_c_, respectively, where they are not able to infect the vectors. Seroconversion and infectivity are characterized by two late asymptomatic stages (A_dr_ and A_dd_ for dogs, and A_cr_ and A_cd_ for cats), and occur at a rate **β**. Fractions **f**_**dr**_ and **f**_**cr**_ (for dogs and cats, respectively) of infected individuals will move to the A_dr_ and A_cr_ compartment and recover spontaneously. Other minor fractions **f**_**dd**_ and **f**_**cd**_ (for dogs and cats, respectively) will move to the asymptomatic stages A_dd_ and A_cd_ and will develop symptoms. The A_dr_ and A_cr_ individuals stay at this compartment until their humoral immunity response becomes low or reaches zero, and at a rate **δ** (**δ**_**d**_ and **δ**_**c**_, for dogs and cats, respectively) they go to the recovered compartment (R_d_ or R_c_). The A_dd_ and A_cd_ individuals become sick at a rate **ϕ** (**ϕ**_**d**_ and **ϕ**_**c**_, for dogs and cats, respectively), going to the D_d_ or D_c_ compartment, where they can still infect the vector. A fraction of the sick individuals dies due to disease progress, and another small fraction recovers spontaneously, at a rate **σ** (**σ**_**d**_ and **σ**_**c**_, for dogs and cats, respectively), going to the recovered compartment (R_d_ or R_c_), where their humoral immune response becomes low or reaches zero. When these recovered individuals lose their cellular immune response, at a rate **γ**, they become susceptible again, going to the S compartment (Eqs 10–15 for dogs, and 16–21 for cats).

Dogs

$$\frac{dS_{d}}{dt}=-S_{d}V_{3}ba_{d}\rho_{d}+R_{d}\gamma_{d}+{\text{Σ}}_{d}$$ (10)

$$\frac{dL_{d}}{dt}=S_{d}V_{3}ba_{d}\rho_{d}-L_{d}\left(\beta f_{dr}+\beta f_{dd}+\mu_{d}\right)$$ (11)

$$\frac{dA_{dr}}{dt}=L_{d}\beta f_{dr}-A_{dr}\left(\delta_{d}+\mu_{d}\right)$$ (12)

$$\frac{dA_{dd}}{dt}=L_{d}\beta f_{dd}-A_{dd}\left(\phi_{d}+\mu_{d}\right)$$ (13)

$$\frac{dD_{d}}{dt}=A_{dd}\phi_{d}-D_{d}\left(\sigma_{d}+\alpha_{d}+\mu_{d}\right)$$ (14)

$$\frac{dR_{d}}{dt}=D_{d}\sigma_{d}+A_{dr}\delta_{d}-R_{d}\left(\gamma_{d}+\mu_{d}\right)$$ (15)

Cats

$$\frac{dS_{c}}{dt}=-S_{c}V_{3}ba_{c}\rho_{c}+R_{c}\gamma_{c}+D_{c}\alpha_{c}+{\text{Σ}}_{c}$$ (16)

$$\frac{dL_{c}}{dt}=S_{c}V_{3}ba_{c}\rho_{c}-L_{c}\left(\beta f_{cr}+\beta f_{cd}+\mu_{c}\right)$$ (17)

$$\frac{dA_{cr}}{dt}=L_{c}\beta f_{cr}-A_{cr}\left(\delta_{c}+\mu_{c}\right)$$ (18)

$$\frac{dA_{cd}}{dt}=L_{c}\beta f_{cd}-A_{cd}\left(\phi_{c}+\mu_{c}\right)$$ (19)

$$\frac{dD_{c}}{dt}=A_{cd}\phi_{c}-D_{c}\left(\sigma_{c}+\alpha_{c}+\mu_{c}\right)$$ (20)

$$\frac{dR_{c}}{dt}=D_{c}\sigma_{c}+A_{cr}\delta_{c}-R_{c}\left(\gamma_{c}+\mu_{c}\right)$$ (21)

#### Rabbits and hares

These hosts are born as susceptible (S_r_ and S_l_ for rabbits and hares, respectively) at a *per capta* birth rate of **Σ**_**r**_ or **Σ**_**l**_, which consists of the natural death rates (**μ**_**r**_ and **μ**_**l**_, for rabbits and hares, respectively) of all rabbit populations, assuming a constant population size. The susceptible individuals are infected at a rate **baρ**, where **b** is the probability of becoming infected when bitten by an infective sandfly (V_3_), **a**_**r**_ and **a**_**l**_ are the daily averages of biting rates on rabbits and hares, respectively, and **ρ**_**r**_ and **ρ**_**l**_ are the density of the vectors per rabbit and hare, respectively. After the infection, these hosts get in an asymptomatic stage being able to infect the vectors (I). They don’t develop symptoms [15,18] and stay in this compartment for more than one season of infection, as observed in hares [15]. After this stage they move to the recovered compartment (R_r_ or R_l_) at a rate **σ (σ**_**r**_ and **σ**_**l**_, for rabbits and hares, respectively). When these recovered individuals lose their cellular immune response, they become susceptible again, going to the S_r_ and S_l_ compartment at a rate **γ (γ**_**r**_ and **γ**_**l**_, for rabbits and hares, respectively) (Eqs 22–24 for rabbits and 25–27 for hares).

Rabbits

$$\frac{dS_{r}}{dt}=-S_{r}V_{3}ba_{r}\rho_{r}+R_{r}\gamma_{r}+\left(I_{r}+R_{r}\right)\mu_{r}$$ (22)

$$\frac{dI_{r}}{dt}=S_{r}V_{3}ba_{r}\rho_{r}-I_{r}\left(\sigma_{r}+\mu_{r}\right)$$ (23)

$$\frac{dR_{r}}{dt}=I_{r}\sigma_{r}+R_{r}\left(\gamma_{r}+\mu_{r}\right)$$ (24)

Hares

$$\frac{dS_{l}}{dt}=-S_{l}V_{3}ba_{l}\rho_{l}+R_{l}\gamma_{l}+\left(I_{l}+R_{l}\right)\mu_{l}$$ (25)

$$\frac{dI_{l}}{dt}=S_{l}V_{3}ba_{l}\rho_{l}-I_{l}\left(\sigma_{l}+\mu_{l}\right)$$ (26)

$$\frac{dR_{l}}{dt}=I_{l}\sigma_{l}+R_{l}\left(\gamma_{l}+\mu_{l}\right)$$ (27)

#### Vectors

The vectors are born as susceptible (V_1_), at a rate **Σ**_**f**_, which consists of the sum of natural death rates (**μ**_f_ and **μ**_s_, for V_2_ and V_3_, respectively) of all vector populations, assuming a constant population size. Vectors get infected when biting the infectious hosts (D_v_, D_ct_, A_dr_, A_dd_, A_cr_, A_cd_, I_r_, and I_l_) at different rates which depend on the fraction of the sandflies that acquire the infection from each host **(c**_**h**_**—**humans, **c**_**d**_**—**dogs, **c**_**c**_**—**cats, **c**_**r**_**—**rabbits, and **c**_**l**_**—**hares) and on the daily average of bites on each host (**a**_**h**_**—**humans, **a**_**d**_**—**dogs, **a**_**c**_**—**cats, **a**_**r**_**—**rabbits, and **a**_**l**_**—**hares), which is based on 1.16 per gonotrophic period [19]. The infected flies (V_2_), when become infective, go to the compartment V_3_, at a rate **τ**, given by the extrinsic incubation period. The vector life expectancy is defined by **μ**_**v**_, **μ**_**f**_, and **μ**_**m**_, for each of its phases (V_1_, V_2_, and V_3_, respectively) (Eqs 28–30). The life expactancy for other phlebotomines was found to be as 8, 5, and 6.6 days (*Lutzomyia longipalpis*, *Pintomyia ficheri*, and *Migonemyia migonei*, respectively) after the first blood feeding [20]. There are no data about the life expectancy of *P*. *perniciosus*, thus to fit the model to represent the proportion of infected by *Leishmania* host in this outbreak we estimated this value at around the values for other vector species. Hence, we take life expectancy of V_2_ plus V_3_ together to be equal to seven days.

$$\frac{dV_{1}}{dt}=-V_{1}\left(\left(A_{hr}+A_{hd}+D_{v}+D_{ct}\right)c_{h}a_{h}+\left(A_{dr}+A_{dd}+D_{d}\right)c_{d}a_{d}+D_{c}c_{c}a_{c}+I_{l}c_{l}a_{l}+I_{r}c_{r}a_{r}\right)+V_{2}\mu_{f}+V_{3}\mu_{s}$$ (28)

$$\frac{dV_{2}}{dt}=V_{1}\left(\left(A_{hr}+A_{hd}+D_{v}+D_{ct}\right)c_{h}a_{h}+\left(A_{dr}+A_{dd}+D_{d}\right)c_{d}a_{d}+D_{c}c_{c}a_{c}+I_{l}c_{l}a_{l}+I_{r}c_{r}a_{r}\right)-V_{2}\left(\tau +\mu_{f}\right)$$ (29)

$$\frac{dV_{3}}{dt}=V_{2}\tau -V_{3}\mu_{s}$$ (30)

#### Prevention and control measures focused on dogs

Vaccine: It was assumed that vaccinated animals do not develop infection or infect the vector. Vaccination is applied to seronegative, such as susceptible (S_d_), recovered (R_d_), and newly infected, non-infective (L_d_) dogs, at a rate of **v**. This rate corresponds to the percentage of dogs per year that are intended to receive protection; it was calculated by taking the efficacy of the vaccine into consideration (Table 1). The loss of vaccine-induced immunity occurs at a rate of **vp** (Eq 7, S1 Text).

**Table 1:** Parameters of the model and its symbols, biological meanings, values, and references.

P	BIOLOGICAL MEANING	VALUE	SOURCE
**HUMANS**			
p_c_	proportion of infections that evolve to CL	0.635	[5]
p_v_	proportion of infections that evolve to VL	0.365	[5]
**1/φ**_h_	Time to appearance of symptoms in humans	2–6 months (PI = 1day-1year)	[60, 61]
**1/α**_h_	Lethality in sick humans	0.0331	[62]
**1/ω**_v_	Period with symptoms of VL	25 days (9–41) + 30 days	[6,61]
**1/ω**_ct_	Symptoms period of CL	109 days (35–183) (até tto) + 15 days (tto)	[6,63]
**1/γ**_v_	Recovery period of VL	10 years	Estimated based on Badaro et al., Carvalho et al., Alvar [64–66]
**1/γ**_ct_	Recovery period of CL	10 years	Estimated based on [67]
**1/σ**_h_	Recovery time of treated individuals	2 years	Estimated based on Carvalho et al., Silva et al., Alvar. [65,66,68]
**1/δ**_h_	Recovery period of humans with asymptomatic VL	22 months	Estimated based on Viana et al. [67]
**f**_hr_	Proportion of humans that develop asymptomatic VL	0.8	[69]
**f**_hd_	Proportion of humans that develop symptomatic VL	0.2	[69]
**μ**_h_	Natural death rate of humans	1/83.3 years^-1^	[70]
**DOGS**			
**1/δ**_d_	Recovery time of dogs with asymptomatic VL	1 year	Estimated based on Fisa et al., Silva et al. [71,72]
**1/γ**_d_	Time to recover from VL	2 year	Estimated based on Garcia et al. and Pozio et al. [73,74]
**1/φ**_d_	Time to appearance of symptoms in dogs	2 months	[75]
**1/σ**_d_	Symptomatic period of VL in dogs	1 year	[74]
**1/α**_d_	Lethality in sick dogs	0.88	[74]
**f**_dr_	Proportion of dogs that develop asymptomatic VL	0.62	[74]
**f**_dd_	Proportion of dogs that develop symptomatic VL	0.38	[74]
**μ**_d_	Natural death rate of dogs	0.067 years^-1^	[76]
**v**	Vaccine coverage	E x Coverage	
**vp**	Period of vaccine protection	1 year	
**E**	Vaccine efficacy	0.70	[41]
**w**	Insecticide impregnated collar coverage	Coverage	
**ψ**	Period of collar protection	6 months	[47]
**mt**	Collar insecticide efficacy	0.55	[47]
**CE**	Collar repellent effect	0.90	[47]
**CATS**			
**1/φ**_c_	Time to appearance of symptoms in cats	2 months	[75]
**1/γ**_c_	Recover period of VL	2 year	Estimated based on Garcia et al. and Pozio et al. [73,74]
**1/σ**_c_	Symptomatic period of VL in cats	1 year	Estimated based on Pozio et al. [74]
**1/δ**_c_	Recovery period of cats with asymptomatic VL	1 year	Estimated based on Fisa et al., Silva et al. [71,72]
**f**_cr_	Proportion of cats that develop asymptomatic VL	0.62	Estimated based on Miró et al. [56]
**f**_cd_	Proportion of cats that develop symptomatic VL	0.38	Estimated based on Miró et al. [56]
**μ**_c_	Natural death rate of cats	0.067 year^-1^	Estimated based on Bouza [77]
**HUMANS, DOGS AND CATS**			
**β**	Latency period in humans, dogs and cats	0.005 days	[78]
**HARES**			
**1/γ**_l_	Recovery period of VL	6 months	Estimated
**1/σ**_l_	Symptomatic period of VL in hares	1 year	Estimated based on Galvez et al. [39]
**μ**_l_	Natural death rate of hares	0.083 year^-1^	[79]
**RABBITS**			
**1/γ**_r_	Recovery period of VL	6 months	Estimated
**1/σ**_r_	Symptomatic period of VL in rabbits	1 year	Estimated based on Galvez et al. [39]
**μ**_r_	Natural death rate of rabbits	0.01 year^-1^	[80]
**VECTORIAL CAPACITY**			
**b**	Rate of infective bites	0.01	[81]
**a**_h_	Daily bites of vector on humans* blood meal rate	60.83*0.03	[17,19,82]
**a**_d_	Daily bites of vector on dogs*blood meal rate	60.83*0.05	[17,19,82]
**a**_c_	Daily bites of vector on cats*blood meal rate	60.83*0.05	[17,19,82]
**a**_l_	Daily bites of vector on hares*blood meal rate	60.83*0.6	[17,19,82]
**a**_r_	Daily bites of vector on rabbits*blood meal rate	60.83*0.6	[17,19,82]
**μ**_v_	Death rate of V2	0.16 days	Estimated
**a**_dc_	Daily bites of vector on collar-wearing dogs*blood meal rate * (1 –CE)	(60.83*0.05)*0.1	
**μ**_s_	Death rate of V3	1 day	Estimated
**τ**	Gonadotrophic period	6 days	[82]
**c**_h_	Proportion of the vectors that acquire infection biting humans	0.6%	[83]
**c**_d_	Proportion of the vectors that acquire infection biting dogs	32%	[15]
**c**_c_	Proportion of the vectors that acquire infection biting cats	4%	[84]
**c**_l_	Proportion of the vectors that acquire infection biting hares	4.7%	[85]
**c**_r_	Proportion of the vectors that acquire infection biting rabbits	1%	[17]
**ρ**_h_	Vector per humans	0.003	Estimate
**ρ**_d_	Vector per dogs	0.1	Estimate
**ρ**_c_	Vector per cats	0.4	Estimate
**ρ**_l_	Vector per hares	2	Estimate
**ρ**_r_	Vector per rabbits	0.5	Estimate

P) Parameter symbols. The parameter “daily bites of vectors per hosts” (**a**) consist in the extrinsic incubation period associated with the blood meal rate of the different host species

Vaccination does not inhibit the development of disease when dogs are vaccinated during the “immunological window” (L_d_) [21]. Therefore, when dogs are vaccinated while in the compartment L_d_, they do not move to the compartment V_d_ but remain in the same compartment, where they go through the natural course of the infection. However, these individuals must be considered in the calculation of the vaccine doses because they are, in fact, vaccinated.

Insecticide impregnated collar: We assumed that all dogs could be collared (asymptomatic or not). The coverage rate of collar is represented by **w** and after six months of use its efficacy is lost (represented by **ψ**), thus the animals return to original compartment, as if without collar (Eqs 8–13, S1 Text).

The collar has two effects, as repellent and insecticide. The first effect induces the reduction of the daily average of vectors bites, represented by **a**_**dc**_ (Eqs 8 and 9 in S1 Text). The second effect causes death of infected and infective vectors (V_3_) that have bitten a collared dog, which is represented by **μ**_**k**_ (mt*a_**dc**_). The naïve vectors (V_1_) that do not die from the insecticide effect go to the infected compartment (V_2_), represented by 1- **mt** (**mt** = vector mortality caused by collar). Thus, the Eqs 28–30 of vectors were modified for the use of collar (Eqs 14–16, S1 Text).

The model with interventions of prevention and control is illustrated in the S1 Fig and represents a part of the model illustrated in the Fig 1.

### Parameters

The parameters were obtained from other published research in the referred region, and some of them were determined aiming the best representation of the 2011 outbreak scenario (Table 1), such as considering the proportion of each population (infected and not infected).

### Simulations

#### Vector control

The use of insecticide with residual activity in the environment influences the parameters such as “density of the vector per host” and “life expectancy of vectors” (infected and non-infected). The use of insecticide in the park area was simulated trough the reduction of the density of vector per hare, rabbit, and cat, and in the urban area were simulated through the reduction of the density of vectors per human, dog, and cat. We consider insecticide reducing these two parameters by 75% (i.e. we subtracted 75% of each parameter in the simulation).

#### Culling hosts

The euthanasia of 100% and 50% of hares and rabbits, by species and both species together were simulated. These simulations were performed with a reproductive rate of 100% and 50%, which indicate that the population size is not exactly constant in this case of culling animals if they are born at a lower reproductive rate and not immediately.

#### Dog with vaccine and insecticide impregnated collar

The simulations of dog vaccination and use of insecticide impregnated collars were based on the efficacy corresponding to the available products in Europe (Table 1) and using the coverages of 50% and 75%.

The model assumes loss and damage of the collars, at a rate of 4.9% (1,796/36,638), as observed by Camargo-Neves, et al [22]. Hence, the coverages of 50 and 75% will be represented as 47.55 and 71.325%, respectively.

#### Sensitivity of the model

The parameters, as “density of the vector per host” and “proportion of the vectors that become infected after biting hosts” were varied by 1% (plus and minus), and we calculated the change in each proportion of infected host in relation of the proportion of infected host without parameter variation. This approach computes an approximate value of the elasticity of each proportion of infected host with regard to the parameter. We also performed global sensitivity analysis to determine the key factors that affect the human leishmaniasis prevalence from the parameters such as “daily biting rate” and “vectors per host” sampled randomly from uniform distribution. “Daily biting” rate parameters were sampled randomly from uniform distribution that ranges from 1 to 100. Similarly, the “vector per host” parameters were randomly sampled from 0.0001 to 10.

## Results and discussion

The infected host populations were affected by the simulated strategies to prevent and control leishmaniasis. Table 2 shows the impact of these strategies on the proportion of infected hosts after the interventions, and with no interventions.

**Table 2:** The influence of the measures in the decrease of the proportion of infected population incidences (infected after/before interventions).

Interventions	cover	HUMANS (Sick %)	DOGS (Seropos %)	CATS (Seropos %)	HARES (Seropos %)	RABBITS (Seropos %)	Protected dogs by vaccine
Population without use of control measures		0.0101	2.36	6.19	60.77	46.54	0
kh+kr	50%	0.0099	2.32	6.09	60.09	40.40	0
	75%	0.0096	2.26	5.96	59.39	34.76	0
kh+kr (50% rr)	50%	0.0016	0.52	1.44	2.80	3.13	0
	75%	0.0002	0.08	0.22	0.20	0.26	0
kh (50% rr)	50%	0.0062	1.53	4.18	3.28	38.79	0
	75%	0.0057	1.39	3.85	0.30	37.44	0
kr (50% rr)	50%	0.0082	2.00	5.35	59.52	5.59	0
	75%	0.0084	1.94	5.20	59.31	1.35	0
Vaccine (Clin Eff = 18.5%)	50%	0.0101	1.53	6.18	60.76	46.51	27%
	75%	0.0101	1.22	6.17	60.75	46.50	39%
Collar	50%*	0.0101	1.46	6.17	60.75	46.50	0
	75%*	0.0100	1.02	6.17	60.75	46.49	0

Cover) Coverage or intensity of the measure effect; Seropos) Seropositives; kh) Culling hares; kr) Culling rabbits; rr) Reproductive rate; Clin Eff) Clinical vaccine efficacy; Seropos) Proportion of seropositive population; Sick) Proportion of sick population;

*At these coverages are being considered the reduction of 4.9% of loss and damage.

The number of human leishmaniasis cases decreased 24.4% (from 160, in 2012, to 39, in 2015) due to the strategies of prevention and control that have been effective [23]. One of these measures was the elimination of the major part of the hares and rabbits inside the park of the outbreak area during this period [12]. We can observe in the present model that culling 75% of both hares and rabbits without immediate reposition of the animals (considering reproductive rate of 50%) is the best measure to reduce the proportion of infected host by *Leishmania*. In this case the infected host populations decreased in a range of 300 times, for hares (seroprevalence from 60.77 to 0.20%), of 30 times, for dogs (seroprevalence from 2.36 to 0.08%), and of 50 times for humans (seroprevalence from 0.0101 to 0.0002%) (Table 2). These proportions of euthanasia simulated here may be feasible in practice, once between December 2011 and February 2013, about 615 rabbits and 1,200 hares were captured in the park, representing a high population density of around 265 hares/km^2^ [14]. Martcheva [24] considered a mathematical model to evaluate the use of control measures for avian influenza, including culling/vaccination of poultry, wearing protective gear for humans and others, and also found that culling the main host (poultry in this case) without re-population presented the best strategy, and the human cases reduced 22%. Thus, we may suggest that our result may be applicable in other similar outbreak situations.

In addition, the difference between culling hares and rabbits without immediate reproduction in a coverage of 75% leads to an impact of more than six times in decreasing the proportion of all infected hosts compared to a coverage of 50% (Table 2), proving to be an efficacious measure to control the disease. Likewise, the number of these wild hosts may have been reduced due to this previous elimination, which in some cases can also decrease the basic reproductive rate and, consequently, the population with time. In the case of wild boars (*Sus scrofa scrofa*), the elimination of 35% of the adult population decreased the growth rate to negative, eliminating the population [25]. On the other hand, eliminating badgers (*Meles meles*) to control *Mycobacterium bovis* resulted in disruption of social groups, due to their territorial behavior, increasing the disease transmission [26]. Territorial behavior were not observed in hares [27] and this could be a limiting factor for the disease spread in the outbreak area. According to Desbiez et al. [25], the knowledge of the survival and reproduction rates, as well as the characteristics of growing and reduction of the population is the first step to its control.

In our model when the reproductive rate of hares and rabbits were 100%, representing immediate replacement of animals, the results were highly unfavorable for the human leishmaniasis control because all the euthanized animals were immediately born as susceptible, providing the vector feed source in the environment. However, when many animals are eliminated from one population, it is possible that the replacement does not occur immediately. The population of Hainan hare (*Lepus hainanus*), an endemic species of the Hainan Island, China, for example, was reported to have suffered greatly from habitat alteration, in addition to hunting pressures [28].

**Fig 2:**
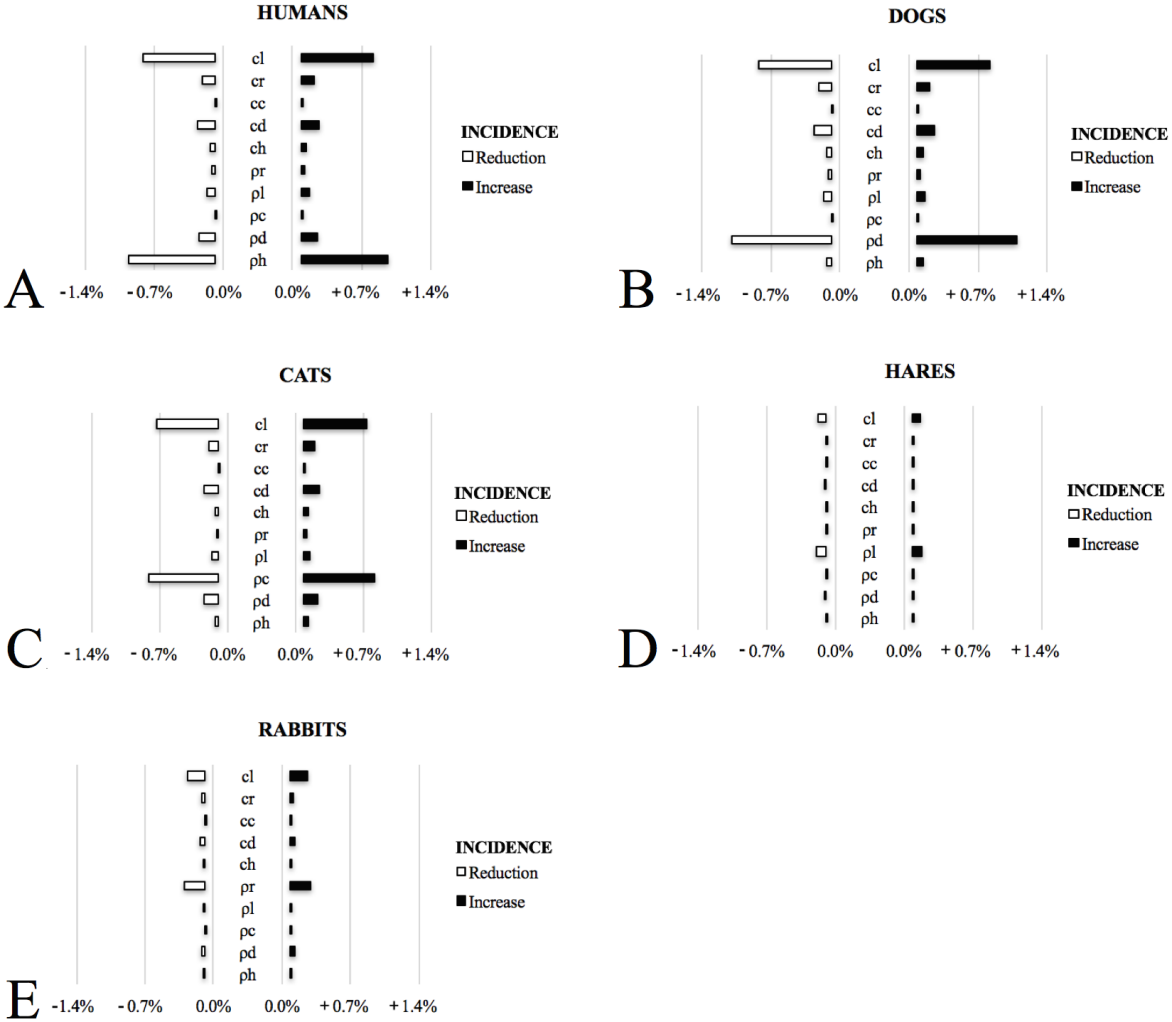
Average variations in sick humans (A), and seroprevalence of dogs (B), cats (C), hares (D), and rabbits (E) in response to the 1% parameter variations. cl, cr, cc, cd, ch) Rate of the vector acquiring infection biting hares, rabbits, cats, dogs, and humans, respectively; ρl, ρr, ρc, ρd, ρh) Density of the vector per hares, rabbits, cats, dogs, and humans, respectively; Reduction) Effect on infected hosts when the parameters are decreased; Increase) Effect on infected hosts when the parameters are increased.

The results of our study showed that culling only hares was more effective than culling only rabbits to decrease the disease proportion of infected hosts (Table 2). Furthermore, the impact of parameters related to the vector contact with hares resulted in a higher influence on the proportion of infected animal hosts and humans. Fig 2 represents the elasticity of parameters. As an example, the reduction or increase of the parameter “proportion of vectors that become infected after biting hares” with 1% changes the dog seroprevalence from 2.36% (without interventions—Table 2) to 2.34% and 2.38% respectively, which means an average impact of 0.85% for minus and plus, respectively. Hence, the 1% variation of the parameter “proportion of vectors that become infected after biting hares” affected the infected populations of human, cat, and rabbit at an average rate of 0.83, 0.73, and 0.2%, respectively. The parameter “density of the vectors per hare” when varied 1% affects these populations at average rates ten times lower (Fig 2). We suggest that this is because of a high number of hares in the environment and also of their higher proportion of the infected population than other hosts. In addition, the parameters related to vector and other hosts do not affect at high intensity the infected population of hare (small bars in Fig 2D), demonstrating that the most important host is the hare, as previously suggested by [13,15], and not the other host species.

Although the elimination of only hares showed better results than eliminating only rabbits, the culling of both hosts generated best results, also due to the vector and the rabbit behavior, such as (1) easy-to-access blood source for the sand flies, and (2) by the fact that rabbits make burrows, which are well-known breeding sites for sandflies, favoring the large increase of these vector densities [29]. According to Flux & Aneermann [27] and Gibb [30], the organic matter of plant origin available in the rabbits feces accumulated inside the burrows, represents an ideal environment for the growth of phlebotomine larvae [31]. Finally, another important factor is that the significant reduction of the hares population due to culling, in the green park close to the urban area of the outbreak, may have influenced the sandflies to use rabbits as a main source of blood for feeding, because they occupy the same habitat, and hence increasing the sandflies quantity [17].

The European hare inhabits vast areas of central Europe [32], constituting a potential reservoir for *L*. *infantum*. Moreover, translocation of hares at a national scale is frequent, contributing to spread of the disease when they are infected [33]. This is one more reason for these animals to be culled for control of the leishmaniasis in Southeast Madrid outbreak. In addition, the human disease prevalence was reduced using this strategy with great impact in the control of the disease in other hosts, as also observed in the present study. Although, euthanasia of infected animals is a questionable action in some countries, in Spain the pros and cons of the issue were considered carefully. It was established that hares were a pest and a public health problem, hence the need to be eliminated. In Brazil, for example, the euthanasia of seropositive dogs for controlling of leishmaniasis is very criticized on ethical grounds, mainly by owners and animal protector societies [34]. There is a study showing that this activity can produce psychological damages on veterinary medicals and research technicians by the demands to euthanize healthy animals and the need to face the moral stress [35].

In the Cochrane Review, the use of insecticide showed the most results in reducing the phlebotomine sandfly population and preventing leishmaniasis [36]. From a theoretical perspective, significant advances were made by Macdonald, who proposed that the most effective control strategy against vector-borne infections is to kill adult mosquitoes [37]. In our model, we found that the use of insecticide with residual action in the park and the urban area decreased the proportion of infected animals by *Leishmania* of all hosts (of park and urban area) (Table 3). However, the incidence reduction occur with higher intensity in the area where the measure was applied. Nevertheless, in these simulations it was not possible to reduce the vector life expectancy separately for each area (park and urban), because they are specific parameters for vectors of all areas (**μ**_**v**_, **μ**_**f**_, and **μ**_**m**_, for each of its phases: V_1_, V_2_, and V_3_, respectively). Consequently, decreasing these parameters will affect the vector of both areas, and the intention here is to understand the insecticide action in the different areas. Differently, the parameter “density of vector per hosts” is specific for each host, so we can simulate varying it for hosts of one or the other location. Therefore, we also simulated the use of insecticide considering only the reduction of density of the vector per host, excluding the parameter “life expectancy of vector” (Table 3). It was possible to see that the reduction of the vector density per host of the park (hare, rabbit and cat) decreased the infected population of all hosts (of the park and urban area). On the other hand, the reduction of the vector density per host of urban area (humans, dogs and cats) reduced only their infected populations, although at higher intensity. Regardless, we suggest that the insecticide use for control of the disease need to be applied in all areas of the outbreak, not only in the areas where the main host (hare) resides or areas with high vector quantity (as the park). However, we recognize that vector control by destroying sandflies breeding sites, and through the use of insecticide products, is one of the most difficult actions to succeed, as previously reported by the Government of Madrid [38].

**Table 3:** The influence of insecticide of residual action in decreasing the proportion of infected population.

Interventions	usage	HUMANS (Sick %)	DOGS (Seropos %)	CATS (Seropos %)	HARES (Seropos %)	RABBITS (Seropos %)
Population without use of control measures		0.01	2.36	6.19	60.77	46.54
Insec. Park	75%	0.0074	1.77	3.75	56.14	37.04
Insec. Urban	75%	0.0057	1.39	3.86	58.87	42.30
dvec/h Park	75%	0.0098	2.31	4.79	58.60	41.74
dvec/h Urban	75%	0.0076	1.80	4.89	60.76	46.51

Usage) Intensity of the measure effect; Insec. Park) Reduction of density of vector per host of park and life expectancy of vector; Insec. Urban) Reduction of density of vector per host of urban area and life expectancy of vector; dvec/h) Reduction of density of the vector per hosts; City) The hosts are the humans, dogs and cats; Park) The hosts are hares, rabbits and cats; Seropos) Proportion of seropositive population; Sick) Proportion of sick population.

The canine VL prevalence in the Madrid region is about 6% [23,39], but in the outbreak zone it was even lower [6]. This condition may be explained by the high protection level of the animals, since their owners keep them in closed sheds and use insect repellents for dogs, such as insecticide impregnated collars and insecticide topic products [40]. We fit the model based on this low canine prevalence, and this probably explains the low influence of the measures focused on dogs, and the vector-dog contact on the disease dynamics. The variation of the parameters related to the vector-dog contact do not present a high impact on the other hosts prevalences (Fig 2), as well as the use of measures focused on dogs (insecticide-impregnated collar and vaccine), which affect only the proportion of infected dog (Table 2). The low influence of the dogs on the other infected host proportions can be also explained by previous studies. Researchers from the Salud Madrid [38] found that dogs were not acting as main reservoirs in the human outbreak that began in 2010, in Spain. The increase of leishmaniasis human cases did not correspond to the proportion of dogs infected by *Leishmania* [38].

When the seronegative dogs are vaccinated at a 50% or 75% coverage, almost only the seroprevalence of dog is affected, decreasing to around 1.53% and 1.22% respectively. Differently, when the collar is applied to 50% and 75% of the dogs, the seroprevalences of the dogs showed more reduction, as to 1.46% and 1.02%, respectively (Table 3), even considering loss and damage rate of 4.9%. Although these two measures exhibited distinct efficacies in reducing the canine seroprevalence, it is important to highlight that the coverage rates of each of these measures were different because the vaccine is applied only in seronegative dogs, and the collar in all of them. In the model we considered the vaccine efficacy as 18.5% [41], corresponding to protection from the developing of infection, however the vaccine efficacy in the prevention of clinical signs is higher, as 68.4%, and hence the level of infectivity to the vector is lower because it is corresponding to the progressive forms of the disease. So, the vaccine demonstrated more epidemiologic advantages besides decreasing the number of infected dogs, such as reducing infective vector numbers and mainly generating herd immunity. The effects of both measures are positive for the disease control, since canine VL is an economically important disease in Europe. Costs are related to direct and indirect factors, such as diagnostic, therapy, lifelong follow up of clinically affected animals, and also to the periodical screening for preventive measures [42]. In our model, we considered the efficacy of the available vaccine in Europe, which actually has additional and important benefits that block the progress of the disease and decrease the infectiveness of dogs to sandflies [43]. Sevá et al. [16] also evaluated the use of impregnated insecticide collar and vaccination in dogs to control leishmaniasis in Brazil, and their impact on reducing the human cases were higher, however in Brazil dogs were considered the main reservoir of the disease agent. The use of deltamethrin-impregnated collar at high rate of dogs in Italy promoted a reduction of 50% in the incidence of canine VL after the first year and 98% after the second year [44].

Solano-Gallego et al. [40] found that the best tool to prevent canine leishmaniasis is the individual use of synthetic pyrethroids on dogs. In field trials, the protection with these products ranged from 50% to 100% during two transmission seasons and the large-scale use might have had a significant impact on the dog population [45,46]. In Brazil, insecticide impregnated collars were applied in all domestic dogs of one municipality, and both human cases and dog disease incidences reduced [47], but no other hosts were investigated. In the present study the parameter “density of the vectors per dog” is the one that represents the most significant impact on the dog populations, decreasing or increasing the seropositive animals at an average of 1.1% when reduced or increased 1%, respectively (Fig 2B). According to García-Martínez & Bernal [48], the chance of dogs becoming infected can increase if insecticides are not used throughout the entire sandfly season. Taking this into account, we have simulated the model using insecticides constantly during the year and with immediate reposition when its efficacy is lost.

According to García-Martínez & Bernal in an endemic area, the use of isolated regular insecticides for a long time has not reduced the canine VL seroprevalence. However, a combination of insecticide impregnated collars (Scalibor^®^) and pour-on pipettes (Advantix^®^) on dogs has resulted in a significant seroprevalence reduction. Even though, on the one hand the use of two antiparasitic formulations concomitantly seems to be more effective for the disease control, on the other hand, it may increase the risk of pyrethroid toxicity. Caution for dogs health safety is required when this option is considered [48].

Foroughi-Parvar & Hatam [9] stated that vaccines are the best approach to employ a convenient and efficacious method for the control of zoonotic VL. The main goal of Leishmaniasis vaccination is to protect against clinical infection and to block the disease progression. In Europe, the CaniLeish^®^ vaccine has been available [41], and it is currently used along with pyrethroids for individual protection as an integrated prevention strategy [40]. The WHO recommends that a sandfly control has to involve more than one method in an integrated vector management approach [1]. We did not simulate more than one measure at a time, with the intention to understand the efficacy of each of the prevention and control strategies.

Control strategies were implemented in cat colonies and street dogs in the Madrid outbreak region since 2010. A total of 1,732 and 2,272 animals were captured, respectively [23]. However, it is difficult to recognize the real effect of these measures, because other strategies to prevent and control the leishmaniasis outbreak have been applied, with great efficacy, reducing significantly the number of cases [23]. In our study, we observed that the vector contact with both cats and dogs does not represent significant impact on the proportion of other infected hosts, and only on their own infected populations. The parameters “proportion of vectors that become infected after biting a cat” and “density of vectors per cat” when varied in 1% affect the infected cat population at a rate of 0.73% and 0.83%, respectively (Fig 2C).

In the same way the vector contact with humans affects only the vector and human infected populations. The parameters “proportion of vectors that become infected after biting a human” and “density of vectors per human” when varied with 1% affect the sick human population in at a rate of 0.06% and 1.01%, respectively (Fig 2A). Thus, the human protective measures, such as topic repellent and insecticides in houses, are also important to control the human cases in this outbreak.

**Fig 3:**
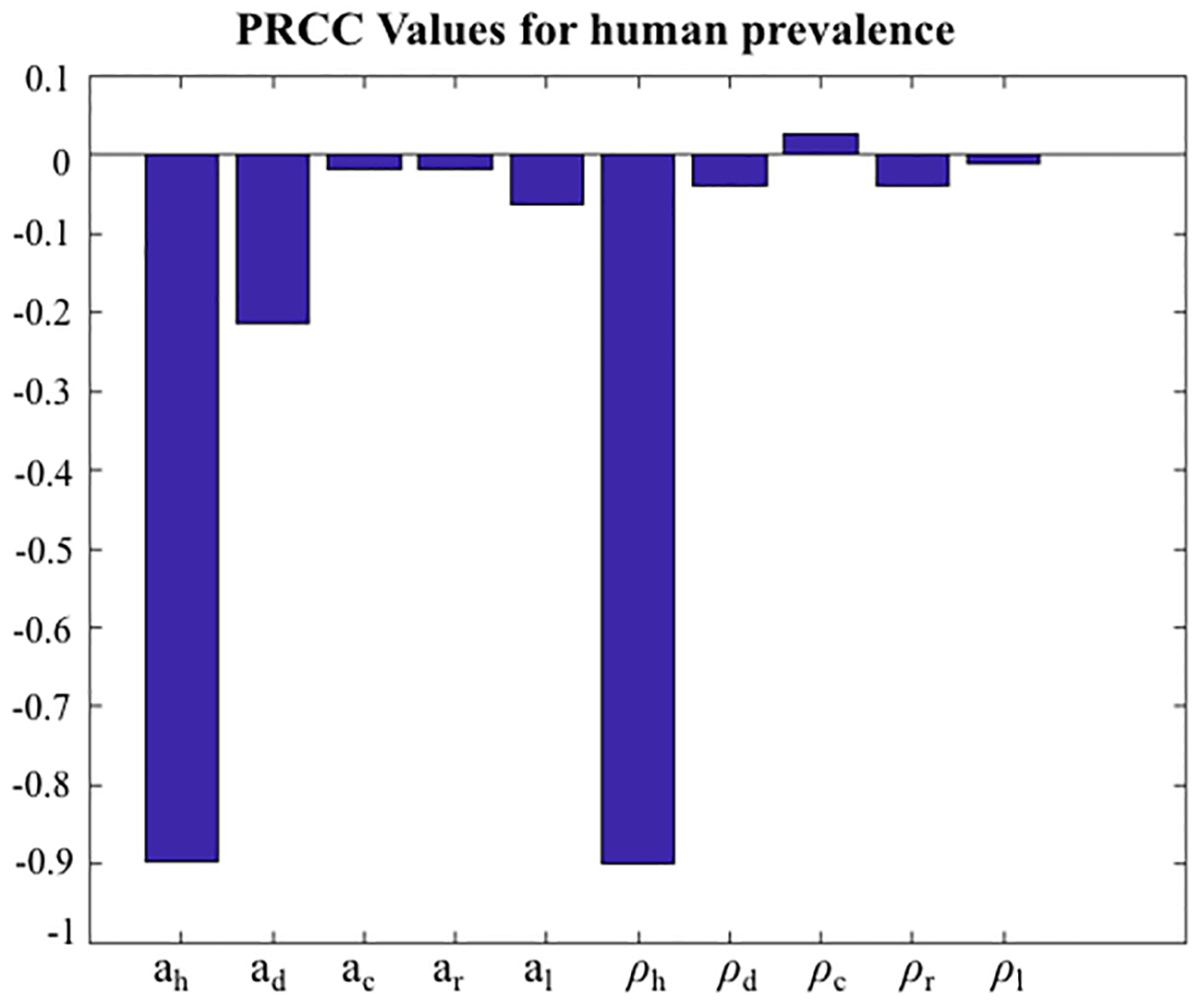
Global sensitivity analysis and its partial rank correlation coefficients (PRCC) of “Daily biting” and “vectors per hosts” parameters. “Daily bites of vector on hosts” rate (**a**_h_, **a**_d_, **a**_c_, **a**_r_ and **a**_l_) and “vectors per host” rate (**ρ**_h_, **ρ**_d_, **ρ**_c_, **ρ**_r_ and **ρ**_l_), following the sequence of hosts: human, dog, cat, rabbits and hares.

**Fig 4:**
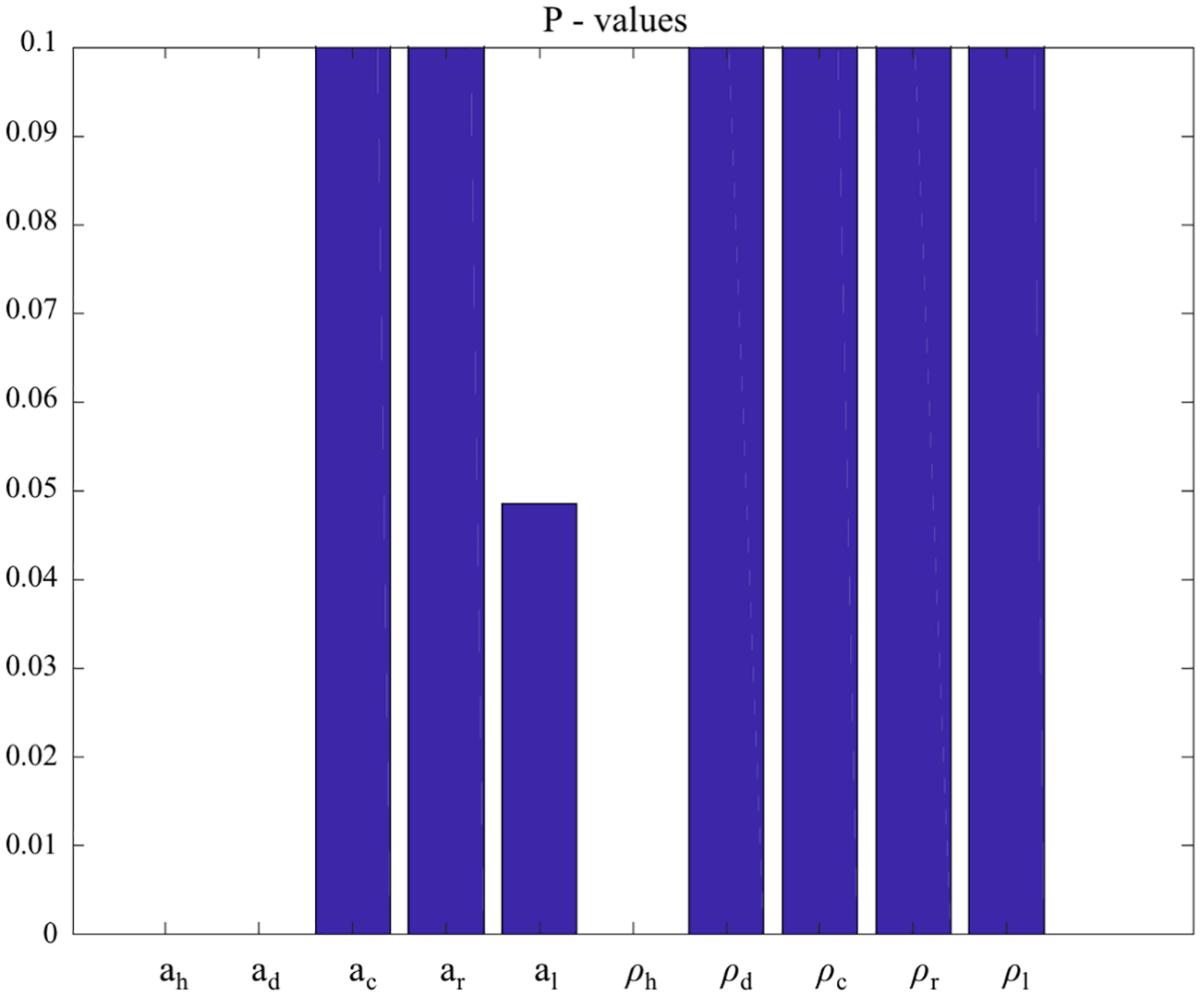
Global sensitivity analysis and its p-values of “Daily biting rates” and “vectors per host” parameters. “Daily bites of vector on hosts” rate (**a**_h_, **a**_d_, **a**_c_, **a**_r_ and **a**_l_) and “vectors per host” rate (**ρ**_h_, **ρ**_d_, **ρ**_c_, **ρ**_r_ and **ρ**_l_), following the sequence of hosts: human, dog, cat, rabbits and hares.

Regarding the global sensitivity analysis to determine the key factors that affect the human leishmaniasis prevalence; we performed partial rank correlation coefficient (PRCC) analysis. The PRCC and their p-values are given in Figs 3 and 4 respectively. The most sensitive parameters were “daily biting rate on humans” and “vector density per human”, represented by the larger bars in the Fig 3 and the minor or absent bars in Fig 4, corresponding to low p-values. As seen from the Fig 4, the daily biting rates for dogs and hares also have significant impact on the output. It is important to understand that the parameters **a**_h_, **a**_d_, **a**_c_, **a**_r_ and **a**_l_ represents daily bites and blood meal rates. However, we are considering the blood meal rate fixed, since the vector bites once per gonotrophic cycle. Thus when these parameters are varied it means that only the blood meals rates are changed.

As we have already discussed, the parameter “vectors per dog” represents significant impact only for the infected dog population (Fig 2B). This value can vary a lot, e.g. 0.0069 in Italy [49] and 1.94 in Brazil [16]. The value estimated by us represents the dog seroprevalence in the outbreak. Once this value does not affect the human prevalence, as the global sensitivity analysis suggests (Figs 3 and 4), our conclusions are also not affected since we are focused on prevention and control of human leishmaniasis, due to the public health perspective of our article.

**Fig 5:**
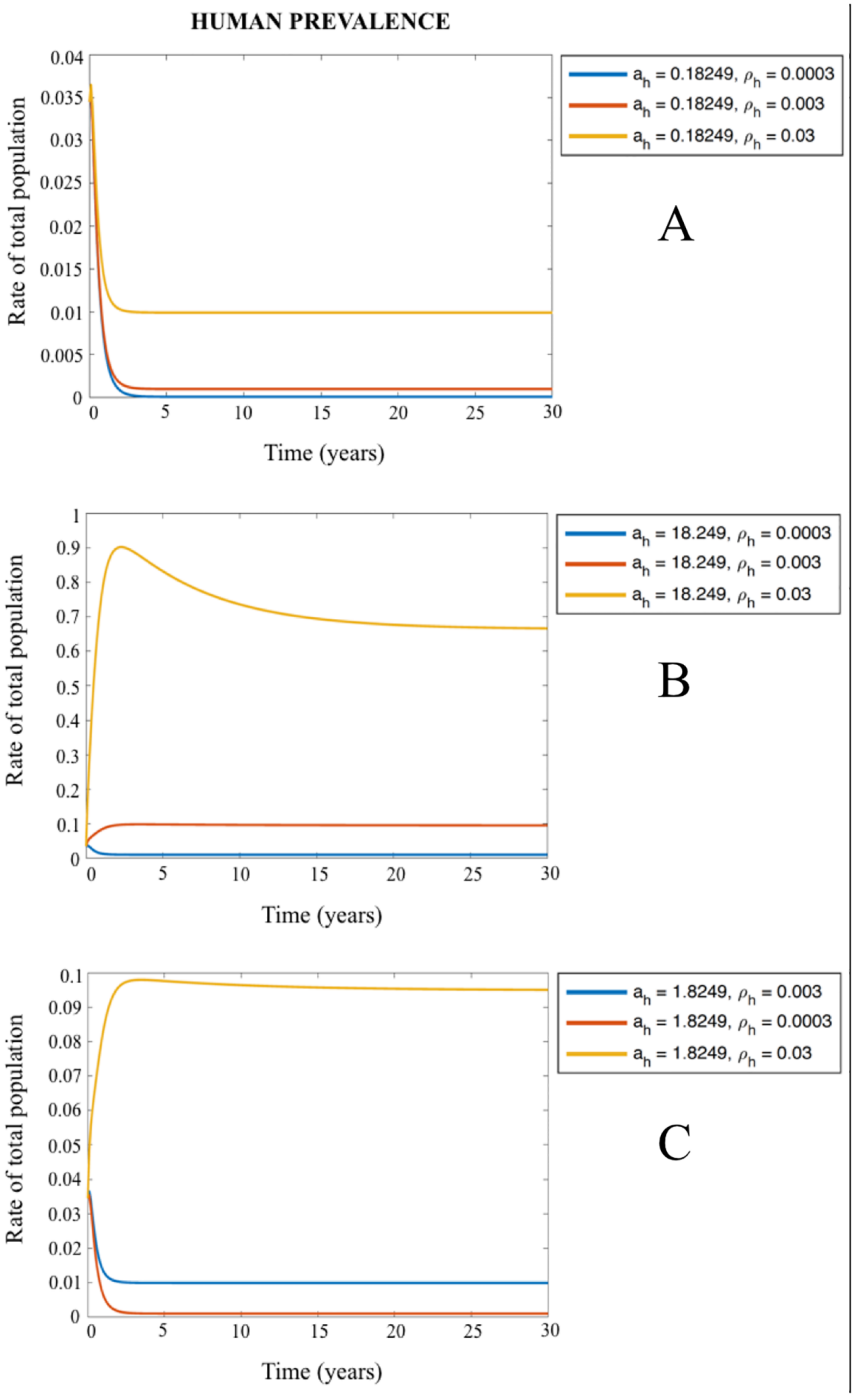
Human prevalences according the variations of the parameters “daily biting rate in humans” (a_h_) and “vector density per human” (ρ_h_). A) a_h_ fixed at 1.8249 and **ρ**_h_ varied from 0.03 to 0.0003; B) a_h_ fixed at 0.18249 and **ρ**_h_ varied from 0.03 to 0.0003; and C) a_h_ fixed in 18.249 and **ρ**_h_ varied from 0.03 to 0.0003.

Since the parameters as “daily biting rate on humans” and “vector density per human” were observed as the most sensitive in the global analysis, we also simulated their variations to understand their impact on the human prevalence. These simulations were done in order to check if the estimated values for them were representing the outbreak. The fixed parameters used in the remainder of the article (see Table 2) are a_h_ = 1.8249 (60.83*0.03) and **ρ**_h_ = 0.003, and we varied both parameters with 1 order of magnitude less and more, and plotted all their combinations. In Fig 5 the simulations show that varying these both values human prevalence varies much, such as between 0.5 and 10 times. The only exception occurs when both parameters are varied together while their product remains fixed (such as ah = 18.249 and **ρ**_h_ = 0.0003 or ah = 0.18249 and **ρ**_h_ = 0.03). This happens because the two parameters enter as a product in the model and we cannot identify them based on the prevalence data. In this case biological reality has to be used to rule out extraneous combinations.

Leishmaniasis is a complex disease, and this outbreak specifically occurred in a territory with urban and park areas, complicating the disease control [38]. Although the number of leishmaniasis human cases has been decreasing, it is still higher than expected in that region, and the adopted strategies need to be continued [23]. The success of a control program is reinforced when the people involved understand the need for an intervention, which includes their personal participation, and the surveillance maintenance to prevent the recurrence of transmission [50].

Leishmaniasis is a disease with different expression around the world, with varying *Leishmania*, host and vector species. The mathematical models should consider the biological factors of these species and the disease prevalences of hosts relative to each reality. There are mathematical models for Brazil and Bangladesh, considering their particular characteristics, which could be applied in similar scenarios [16,51–53]. However, the present study is the first mathematical model based on parameters related to the scenario of the Spain outbreak, which considers the various hosts and vector in an urbanized leishmaniasis.

The present model was implemented according to the data available in the literature, except for some parameters related to the vectorial capacity and disease dynamics in hosts like hares, rabbits and cats. Data from the vector vary according to its sensitivity to environmental factors. We found out that there is a need for information about the sand fly population and the vector behavior [31,54]. To estimate the density of the vectors per host, for example, it is necessary to consider the behavior of the host and their exposure level to the vector. Regarding biological features of infected hares and rabbits, their high influence on the leishmaniasis dynamics is considered recent in that area [15,17], hence some data were not evaluated until now. The cats have been found as an important host for the *Leishmania* agent [55][56–59]; however due to its lack of biological data we estimated some characteristics of the disease as being the same as in domestic dogs. Therefore, these parameters were fitted based on the available data and biological concepts, to produce a solution incidence that represents the scenario of zoonotic leishmaniasis in the outbreak from Spain, incorporation the strategies already used for its control. In addition, to improve the comprehension of the dynamics and, consequently, the model, we have performed sensitivity analysis, as presented above. Although some of these parameters, particularly the ones concerning the vector contact with hosts, were identified as sensitive in the present model this does not overrule the validation of the results, and they can be applied to related scenarios to evaluate measures for prevention and control of the disease.

## Conclusions

Different strategies have been adopted to prevent and control leishmaniasis in the Madrid outbreak, such as environmental cleaning, vector control, control and surveillance of hosts, protectors against insects for the hosts, and public education. We observed with this study that the measures with higher efficacy in reducing the leishmaniasis human cases are related to blocking the contact of the vector with wild hosts, and also controlling the vector in the urban area and the park. The disease control in dogs is effective when using both the insecticide-impregnated collars and vaccination, however these measures are not efficacious in controlling the disease in other hosts. In conclusion, the present mathematical model is able to represent the leishmaniasis dynamics in the Madrid outbreak, highlighting the contribution of each prevention and control strategy. If this scenario of containing the parasite of leishmaniasis in the vector and hosts and this characteristic environmental area (urbanization close to parks), is present in other regions, this model could be applied to direct the efforts to prevention and control of the disease and the monitoring of the use of control measures.

## Supporting information

S1 Fig Model of the leishmaniasis dynamic in dogs with and without insecticide impregnated collar. Legend: Vector: V1) Non infected, V2) Infected but not infective; V3) Infected and infective; Dogs: S_d_) Susceptible; L_d_) Latent with visceral leishmaniasis, Ad) Asymptomatic; D_d_) Sick with visceral leishmaniasis; R_d_) Recovered; Dogs with collar: S_dc_) Susceptible; L_dc_) Latent with visceral leishmaniasis, A_ddc_ and A_drc_) Asymptomatic; D_dc_) Sick with visceral leishmaniasis; R_dc_) Recovered; Vaccinated dog: V_d_.
(TIF)

S1 Text Equations of dogs and vectors adapted for using vaccine and insecticide impregnated collar in dogs. (DOCX)
