# Supplementary figures and images for: Efficacies of prevention and control measures applied during an outbreak in Southwest Madrid, Spain
Source: PLoS One. 2017 Oct 13;12(10):e0186372. doi: 10.1371/journal.pone.0186372 (PMC5640254; doi:10.1371/journal.pone.0186372)

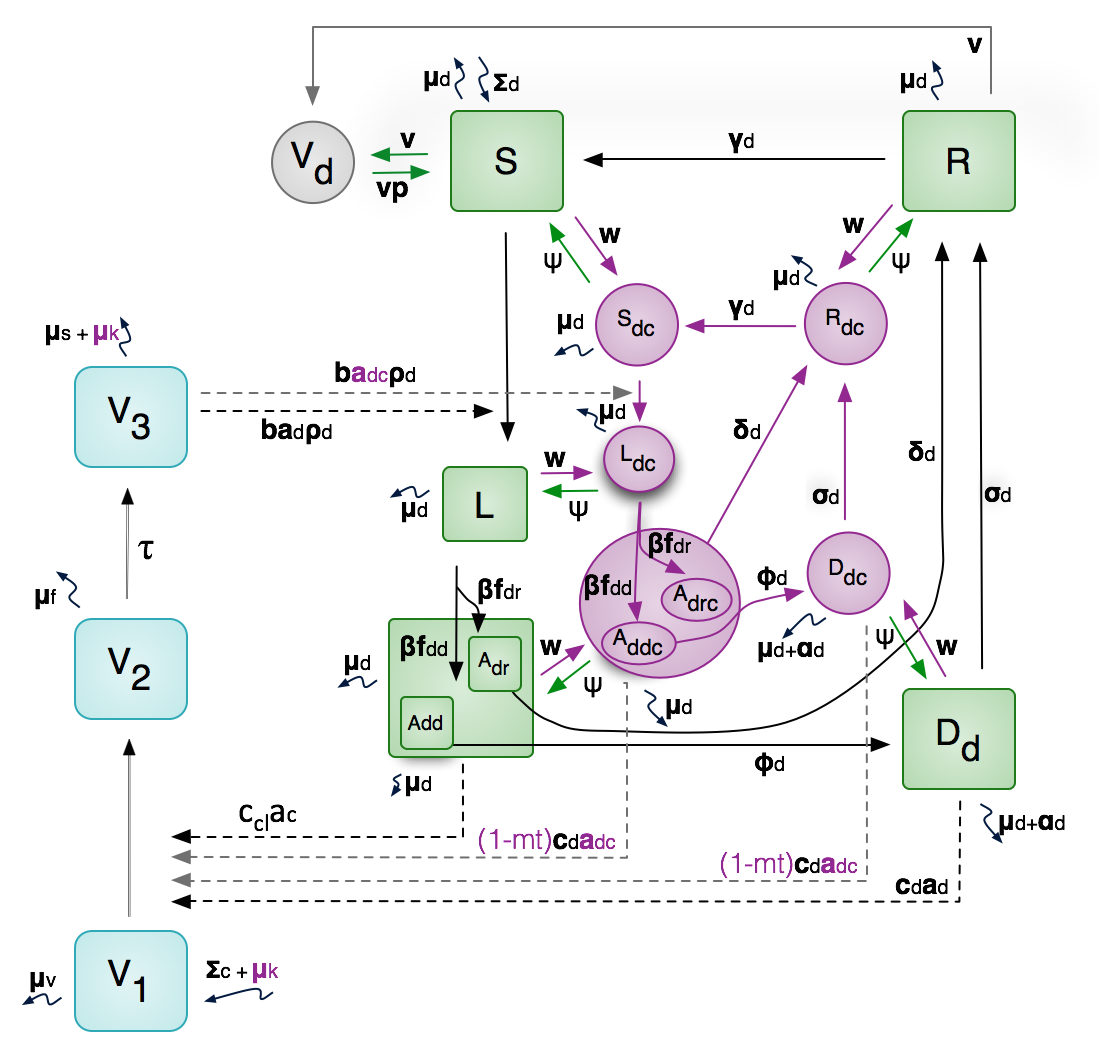

Supplement: S1 Fig — Legend: Vector: V1) Non infected, V2) Infected but not infective; V3) Infected and infective; Dogs: Sd) Susceptible; Ld) Latent with visceral leishmaniasis, Ad) Asymptomatic; Dd) Sick with visceral leishmaniasis; Rd) Recovered; Dogs with collar: Sdc) Susceptible; Ldc) Latent with visceral leishmaniasis, Addc and Adrc) Asymptomatic; Ddc) Sick with visceral leishmaniasis; Rdc) Recovered; Vaccinated dog: Vd. (TIF) [file pone.0186372.s001.tif]
