# Supplementary material for: Efficacies of prevention and control measures applied during an outbreak in Southwest Madrid, Spain
Source: PLoS One. 2017 Oct 13;12(10):e0186372. doi: 10.1371/journal.pone.0186372 (PMC5640254; doi:10.1371/journal.pone.0186372)
Supplement: S1 Text — (DOCX) [file pone.0186372.s002.docx]

Equations of dogs and vectors adapted for using vaccine and insecticide impregnated collar in dogs.

**Dogs**

$\frac{dS_{d}}{dt}= -S_{d}V_{3}ba_{d}\rho_{d}+R_{d}\gamma_{d}+ V_{d}vp-S_{d}v-S_{d}w+S_{dc}\psi+\Sigma_{d}$ (1)

$\frac{dL_{d}}{dt}= S_{d}V_{3}ba_{d}\rho_{d}-L_{d}\left( \beta f_{dr}+\beta f_{dd}+\mu_{d} \right)-L_{d}w+L_{dc}\psi$ (2)

$\frac{dA_{dr}}{dt}=L_{d}\beta f_{dr}-A_{dr}\left( \delta_{d}+\mu_{d} \right)-A_{dr}w+A_{drc}\psi$ (3)

$\frac{dA_{dd}}{dt}=L_{d}\beta f_{dd}-A_{dd}\left( \phi_{d}+\mu_{d} \right)-A_{dd}w+A_{ddc}\psi$ (4)

$\frac{dD_{d}}{dt}=A_{dd}\phi_{d}-D_{d}\left( \sigma_{d}+\alpha_{d}+\mu_{d} \right)-D_{d}w+D_{dc}\psi$ (5)

$\frac{dR_{d}}{dt}=D_{d}\sigma_{d}+A_{dr}\delta_{d}-R_{d}\left( \gamma_{d}+\mu_{d}+v \right)-R_{d}w+R_{dc}\psi$ (6)

**Vaccinated dogs**

$\frac{dV_{d}}{dt}=v\left( S_{d}+R_{d} \right)-V_{d}\left( \mu_{c}+vp \right)$ (7)

**Dogs with insecticide impregnated collar**

$\frac{dS_{dc}}{dt}= -S_{d}V_{3}ba_{dc}\rho_{d}+R_{d}\gamma_{d}+S_{d}w-S_{dc}\psi$ (8)

$\frac{dL_{dc}}{dt}= S_{d}V_{3}ba_{dc}\rho_{d}-L_{d}\left( \beta f_{dr}+\beta f_{dd}+\mu_{d} \right)+L_{d}w-L_{dc}\psi$ (9)

$\frac{dA_{drc}}{dt}=L_{d}\beta f_{dr}-A_{dr}\left( \delta_{d}+\mu_{d} \right)+A_{dr}w-A_{drc}\psi$ (10)

$\frac{dA_{ddc}}{dt}=L_{d}\beta f_{dd}-A_{dd}\left( \phi_{d}+\mu_{d} \right)+A_{dd}w-A_{ddc}\psi$ (11)

$\frac{dD_{dc}}{dt}=A_{dd}\phi_{d}-D_{d}\left( \sigma_{d}+\alpha_{d}+\mu_{d} \right)+D_{d}w-D_{dc}\psi$ (12)

$\frac{dR_{dc}}{dt}=D_{d}\sigma_{d}+A_{dr}\delta_{d}-R_{d}\left( \gamma_{d}+\mu_{d} \right)+R_{d}w-R_{dc}\psi$ (13)

**Vectors when using insecticide impregnated collar**

$\frac{dV_{1}}{dt}= -V_{1}\left( \left( A_{hr}+A_{hd}+D_{v}+D_{ct} \right)c_{h}a_{h}+\left( A_{dr}+A_{dd}+D_{d} \right)c_{d}a_{d}+D_{c}c_{c}a_{c}+I_{l}c_{l}a_{l}+I_{r}c_{r}a_{r}+\left( A_{drc}+A_{ddc}+D_{dc} \right)c_{d}(1-mt)a_{dc} \right)+V_{2}\mu_{f}+V_{3}(\mu_{s}{+\mu}_{k})$ (14)

$\frac{dV_{2}}{dt}= V_{1}\left( \left( A_{hr}+A_{hd}+D_{v}+D_{ct} \right)c_{h}a_{h}+\left( A_{dr}+A_{dd}+D_{d} \right)c_{d}a_{d}+D_{c}c_{c}a_{c}+I_{l}c_{l}a_{l}+I_{r}c_{r}a_{r}+\left( A_{drc}+A_{ddc}+D_{dc} \right)c_{d}(1-mt)a_{dc} \right)-V_{2}\left( \tau+\mu_{f} \right)$ (15)

$\frac{dV_{3}}{dt}= V_{2}\tau-V_{3}(\mu_{s}{+\mu}_{k})$ (16)
